# A web server for predicting inhibitors against bacterial target GlmU protein

**DOI:** 10.1186/1471-2210-11-5

**Published:** 2011-07-06

**Authors:** Deepak Singla, Meenakshi Anurag, Debasis Dash, Gajendra PS Raghava

**Affiliations:** 1 Institute of Microbial Technology, Chandigarh, India; 2 G. N. R. Knowledge Centre for Genome Informatics, Institute of Genomics and Integrative Biology (IGIB), New Delhi, India

## Abstract

**Background:**

The emergence of drug resistant tuberculosis poses a serious concern globally and researchers are in rigorous search for new drugs to fight against these dreadful bacteria. Recently, the bacterial GlmU protein, involved in peptidoglycan, lipopolysaccharide and techoic acid synthesis, has been identified as an important drug target. A unique C-terminal disordered tail, essential for survival and the absence of gene in host makes GlmU a suitable target for inhibitor design.

**Results:**

This study describes the models developed for predicting inhibitory activity (*IC*_*50*_) of chemical compounds against GlmU protein using QSAR and docking techniques. These models were trained on 84 diverse compounds (GlmU inhibitors) taken from PubChem BioAssay (AID 1376). These inhibitors were docked in the active site of the C-terminal domain of GlmU protein (2OI6) using the AutoDock. A QSAR model was developed using docking energies as descriptors and achieved maximum correlation of 0.35/0.12 (r/r^2^) between actual and predicted *pIC*_*50*_. Secondly, QSAR models were developed using molecular descriptors calculated using various software packages and achieved maximum correlation of 0.77/0.60 (r/r^2^). Finally, hybrid models were developed using various types of descriptors and achieved high correlation of 0.83/0.70 (r/r^2^) between predicted and actual *pIC*_*50*_. It was observed that some molecular descriptors used in this study had high correlation with *pIC*_*50*_. We screened chemical libraries using models developed in this study and predicted 40 potential GlmU inhibitors. These inhibitors could be used to develop drugs against *Mycobacterium tuberculosis*.

**Conclusion:**

These results demonstrate that docking energies can be used as descriptors for developing QSAR models. The current work suggests that docking energies based descriptors could be used along with commonly used molecular descriptors for predicting inhibitory activity (*IC*_*50*_) of molecules against GlmU. Based on this study an open source platform, http://crdd.osdd.net/raghava/gdoq, has been developed for predicting inhibitors GlmU.

## Background

Antibiotic resistance has become a major hurdle to overcome bacterial diseases and thus there is always a need to find new drug targets or inhibitors or both. At present very few drugs are available in the market for treatment of *M. tuberculosis *infection as evolution of drug-resistant strains have resulted in little efficacy and some of them have shown undesired side-effects in host [1]. Studies suggest that the prevalence of Multi Drug Resistant tuberculosis (MDR-TB) ranged from 6.7% for three drugs to 34% for four drugs and has caused an annual loss of around $4 - $5 billion [2-5]. Keeping in mind the rapidly changing pathogenesis of this lethal micro-organism, identification of novel inhibitors for recently discovered targets has become pressing need of the hour. GlmU is one such target which is essential for the survival of the pathogen [6,7]. Recent studies on the Mycobacterial proteome using *in-silico *analysis suggested GlmU to be a potential drug target [8]. This protein is a bi-functional enzyme that catalyzes a two steps reaction. Initially, catalytic conversion of glucosamine-1-phosphate to N-acetyl-glucosamine-1-phosphate takes place at the C-terminal domain followed by conversion of N-acetyl-glucosamine-1-phosphate to UDP-GluNAc at the N-terminal domain [9,10]. Though the second step is present in prokaryotes as well as in humans, the first step is present only in prokaryotes [6]. The absence of the first step in human makes it suitable for designing non-toxic inhibitors. The three dimensional structure of the GlmU enzyme has been reported from *Escherichia coli, Mycobacterium tuberculosis, Streptococcus pneumoniae, Haemophilus influenzae, Yersinia pestis *in apo and holo-forms [11-14]. These structures have missing coordinates for the C-terminal intrinsically disordered regions.

The identification of inhibitors using experimental techniques is an expensive and tedious job. Thus, there is need to develop theoretical models for predicting inhibitors against a potential target. In the past, a number of models has been developed using QSAR and docking [12-17] for the identification of novel inhibitors against different bacterial targets. Except KiDoQ [18] and CDD [19] none of them is freely available to the scientific community. KiDoQ is based on prediction of binding affinity against Dihydrodipicolinate synthase (DHDPS) enzyme of *E.coli *while CDD is a collection of compounds and predictive models against *M.tb*. It is important that newly developed models for predicting inhibitors should be made available in the public domain, in order to assist researchers in discovering new drugs against diseases of the poor. In this study, a systematic attempt has been made to address these issues. Firstly, we developed QSAR models using docking energies as molecular descriptors. Secondly, QSAR models were developed using commonly used molecular descriptors calculated using various freeware and commercial software packages. Thirdly, hybrid models were developed using docking energy based descriptors and commonly used molecular descriptors. Finally, a web server has been implemented using the best models developed in this study, hence providing an open source platform to the scientific community for discovering new drugs against bacterial target GlmU protein.

## Methods

### Data set

We retrieved 125 GlmU inhibitors from PubChem Bioassay AID-1376 [20,21] with known *IC*_*50 *_values against *M.tuberculosis *GlmU. These inhibitors exhibit a wide range of activity (1-9999 μM) and structural diversity (see clustering at 70% in Additional file-1). There were errors in calculating descriptors for 4 molecules and hence a reduced set of 119 molecules was considered for further analysis. After docking these 119 molecules in active site of GlmU protein, 27 molecules have higher energy than substrate. After removing these molecules, we were left with only 92 molecules which were further studied. At the time of QSAR model development, we observed that around 8 molecules acted as outliers. These molecules were also removed which led us to a final dataset of 84 molecules to be used in this study.

### Docking Protocol

#### Blind Docking

In this approach, we performed blind docking against GlmU protein of *M. tuberculosis *using AutoDock [22]. Ideally molecules should be docked against the GlmU_mtb_, but the coordinates available in the Protein Databank (PDB) for full length (residue 1-479) GlmU_mtb _are unliganded and show a disordered loop (N_397 _to R_405_) in the active site. For these reasons, we developed a structural model of GlmU_mtb _protein using Modeller 9v8 based on 3D8V as the basic template [23]. For the missing loop region in 3D8V, GlmU_ecoli _in liganded form (2OI6) was used as template. This was followed by loop refinement and the model with best DOPE score was selected for further studies. We generated a trimeric state of the modeled structure using Matchmaker utility of chimera [24] with 2OI6 as the template for superposition.

#### Site Specific Docking

In this approach, potential inhibitors were docked in the substrate binding site of GlmU_ecoli_. We obtained the structure of GlmU protein of *E. coli *(2OI6) complex with substrates from the PDB. Since we were focusing on the glucosamine-1-phosphate binding pocket, that requires only 2-chain association, dimeric model was used as input for docking studies after removal of hetero atoms. An automated flexible docking approach was carried out to find effective molecule with specific binding using AutoDock.

#### Receptor and ligand preparation

Protein and ligand preparation was performed using the AutoDock and involved the addition of hydrogen atoms, computing charges, merging non-polar hydrogen atoms and defining AD4 atom types to ensure that atom conformed to the AutoDock atom types. A grid was defined using Autogrid feature of the software and docking conformation search was done using a genetic algorithm (GA) procedure with t-step value of 1.8. Default parameters were used for rest of the options.

### Descriptor Calculation

Descriptors are the basis of any QSAR modeling strategy and we calculated descriptors using various software packages. Firstly, V-Life MDS 2.0 software was used to calculate 1576 descriptors comprising of topological descriptors, physiological descriptors etc. Secondly, 178 descriptors were calculated using open source Web-Cdk [25] software based on CDK library. Thirdly, the Dragon [26] software was used for calculating 1665 descriptors. Additionally, we also used docking energy as descriptors for QSAR modeling. Docking of a compound using AutoDock gives 11 types of energy i.e. free energy, VdW + Hbond + desolv Energy, unbound system energy, moving ligand fixed receptor, Electrostatic Energy, Moving Ligand-Moving Receptor, Final Total Internal Energy, Internal Energy Ligand, Internal Energy Receptor and Torsional Free Energy. These different types of energies were used as descriptors for development of the QSAR based model based on algorithm similar to that of KiDoQ.

### Selection of Descriptors

In QSAR modeling, descriptors play an important role and hence selection of highly important descriptors is necessary for building the most efficient QSAR model. To achieve this, we removed descriptors that were invariable and then used the CfsSubsetEval module implemented in the Weka [27] followed by an F-stepping (leave one out) approach. The CfsubsetEval module along with best fit method finds the best descriptors by considering the predictive ability of each descriptor. While in F-stepping approach, each descriptor is removed from the dataset of *n *variable, followed by model building and evaluation. If removal of descriptor decreases the performance it will be added in the next step otherwise it is removed finally from the dataset. For example, we calculated 1576 descriptors using v-life software. For example, we calculated 1576 descriptors using v-life software. After removing the invariable descriptors, we selected best descriptors using CfsubsetEval implemented in Weka and obtained 20 descriptors. In final step, F-step approach was implemented in which each descriptor is removed one by one and model performance is measured and this gave us 5 descriptors. This procedure was also implemented on other software's calculated descriptors.

### QSAR Models

#### SVM based QSAR models

We used Support Vector Machine (SVM) for prediction of GlmU inhibitors. SVM based on statistical and optimization theory, handles complex structural features. SVM^light ^software package has been used to develop SVM based QSAR models. This software is freely downloaded from http://www.cs.cornell.edu/People/tj/svm_light/. The performance of models was optimized using systematic variation of different SVM parameters and kernels.

#### QSAR model using Weka

Weka is a very popular and reliable package widely used in the field of Bioinformatics and Chemoinformatics [27]. It is a collection of machine-learning algorithms and supports several standard features like classification, regression, data preprocessing, and feature selection. Here we used SMOreg (Sequential Minimization Optimization) implemented in Weka to predict inhibitory activity of GlmU compounds. This implementation globally replaces all missing values and transformed nominal attributes into binary ones and also normalizes all attributes.

#### Multiple linear regression based model

MLR is a statistical technique that finds the linear relationship between two or more independent variables and one dependent variable. In this study, we used the commercial the software STATISTICA [28] for implementing MLR for developing QSAR model.

### Evaluation of QSAR models

To evaluate the performance of the QSAR model, we adopted two different procedures. First, Leave One Out (LOOCV) strategy was implemented in which one molecule is taken from the dataset of 84 compounds (mentioned in Development of QSAR Models section) as a test compound and the remaining 83 compounds used for model building. This process is repeated 84 times such that each compound come in test set one time. Once the model was constructed, fitness of model was assessed using the following statistical parameters.

Where x_i _and y_i _represent actual and predicted *pIC*_*50 *_value for the i^th ^compound, N is number of compounds, and x represents the averaged value of the actual *pIC*_*50 *_value for the whole dataset.

Despite this LOOCV strategy, it is very important to use an independent dataset to access overall performance of QSAR model. Thus to evaluate the performance without any bias, we made a random set of 25 compounds as an independent test set and the remaining compounds were used for model development using the LOOCV method. This cycle was repeated about 25 times and predictive r and r^2 ^on training as well as independent sets were observed as shown in Additional file-2.

where SD is the sum of the Squared Deviations between the activities of the test set and mean activities of the training molecules.

## Results

### Similarity Search

Similarity describes how two compounds are structurally similar to each other. Thus if two compounds are highly similar to each other they should have similar chemical as well as biological properties. Using this concept, we tried to find relationship between actual and predicted inhibitory activity values. In order to predict the activity of a compound, we took the average of *pIC*_*50 *_value for all hits (except self hit) that have high similarity with query compound. We used software JC Search [29] for searching similar compounds using different similarity cutoff value. A poor correlation among the actual and predicted *pIC*_*50 *_values was observed, so this was not pursued further.

### Target Structure for Docking

In PDB, a number of crystal structures for *M. tuberculosis *are present but all these structures are found with missing loop in the active site and also in unliganded state. Thus, we modeled only the missing loop portion (G_400_) of *M. Tuberculosis *crystal structure using Modeller 9v8. All the inhibitors were docked against the modeled structure of GlmU with the help of AutoDock using a blind docking approach. The docking energies of each inhibitor were computed to develop a QSAR model. These docking energies were used as descriptors and QSAR model for predicting inhibition activity of inhibitors was developed. We achieved poor correlation r = 0.15 between predicted and actual *pIC*_*50 *_value of inhibitors.

**Figure 1:**
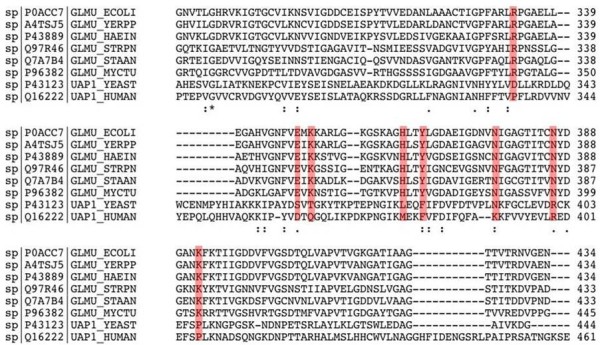
**Multiple sequence alignment of GlmU proteins of different bacterial species and UAP1 proteins of human/yeast using clustalW**. The red color shows conserved residues in active site.

In order to explore alternative strategies, we searched GlmU in other organisms and found a substrate bound crystal structure of GlmU protein in trimeric form in *E.coli*. In order to understand the level of conservation in the glucosamine-1-phosphate active site, we aligned GlmU proteins from the different bacterial species and its homolog (yeast/human) UAP1 using ClustalW [30]. As shown in Figure 1, multiple sequence alignment reveals a high degree of conservation in the active site among the different bacterial species. It was also observed that active site residues of bacterial GlmU have poor conservation with human UAP1 protein. Thus the presence of such a highly conserved set of amino acid residues suggests that inhibitors designed for this site show broad spectrum activity. Site-specific docking was performed against the GlmU_ecoli_. We developed a QSAR model using docking energies as descriptors and achieved correlation of r = 0.37 between predicted and actual inhibition. This correlation is significantly better than the correlation we got in case of blind docking against a modeled structure of GlmU_mtb_. Hence we used site specific docking against a substrate bound GlmU structure of *E. coli *for further study.

#### Evaluation and Validation of Docking Protocol

**Figure 2:**
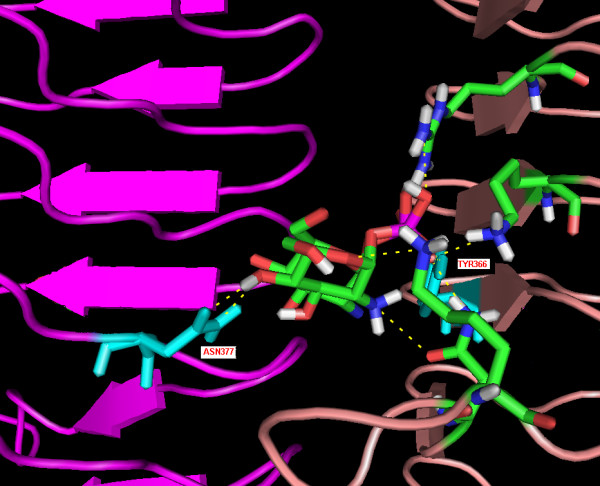
**Shows the superimposed structure of docked substrate over crystal structure**. This figure depict the superimposed structure of docked ligand with crystal structure with active site residues in ball and stick form and hydrogn bond with yelllow colour dashes line. The flexible residues TYR366 and ASN377 are coloured cyan and labeled.

For evaluation of docking protocol, we used the *E.coli *GlmU enzyme crystal structure 2OI6 retrieved from the PDB. We docked glucosamine-1-phosphate into the active site of the protein by making Asn377A and Tyr366C residue flexible. Visually examining the ligand-protein interaction and calculating RMSD between crystal structure and docked structure 0.072 Å was used to validate docking protocol which has been shown in Figure 2.

### QSAR Models

In this study, we developed QSAR models using various algorithms/techniques; this includes techniques like MLR and SVM. It has been observed that MLR based QSAR models perform better or equal to other learning techniques (data not shown). Thus we developed rest of QSAR models using MLR. First, MLR based QSAR model was developed on 84 compounds using five molecular descriptors obtained from V-life descriptors after removing highly correlated descriptors. We obtained correlation r/r^2 ^of 0.75/0.56 between predicted and actual value of p*IC*_*50 *_(Table 1). As shown in Table 1, mean absolute error between predicted and actual inhibitory constant was found to be 0.36. Secondly, QSAR model was developed on same dataset using two best molecular descriptors selected from Web-Cdk descriptors. As shown in Table 1, a correlation r/r^2 ^of 0.56/0.31 with MAE 0.43 was achieved on 84 compounds. In this study, we used docking energies as descriptor and developed QSAR model using these descriptors, similar approach has been used in past for developing KiDoQ [18]. We achieved correlation r = 0.16 using site specific docking and correlation r = 0.15 using blind docking on modeled structure. As evident from Table 1, we got poor correlation r/r^2 ^of 0.35/0.12 using four best docking energies (site specific docking) on *E. coli *structure. The QSAR models based on nine selected descriptors of Dragon perform (correlation r/r^2 ^0.77/0.60) was found to be better than any other model.

**Table 1:** The performance of QSAR models developed based on best descriptors computed using various software and techniques.

Number of Descriptors	Software Packages	R	**R**^**2**^	MAE
5	V-life	0.75	0.56	0.36
2	Web-Cdk	0.56	0.31	0.43
4	Docking Energy Based Descriptor	0.35	0.12	0.44
9	Dragon	0.77	0.60	0.32

One of the important questions is whether selected descriptor used in this study for developing QSAR models also has direct correlation with inhibition constant. For this we computed correlation between selected descriptor and p*IC*_*50 *_as shown in Table 2. It was observed that some of the descriptor even have a correlation higher than 0.5. The quality of descriptor depends on it correlation with inhibition constant, the higher the correlation, better is the descriptor. It is also clear from data shown in Table 2 that performance of QSAR models depended on quality of descriptors. Thus there was a need to develop hybrid model which could utilize best descriptors calculated using various software like Dragon, Web-Cdk, V-life.

**Table 2:** Correlation values for molecular descriptors with p*IC*_*5*__*0 *_value.

WebCdk Descriptors									
**Descriptor**	VCH-4					Wlambda2.unity			
**Correlation**	-(0.50)					0.36			

**Docking based energy descriptors**									

**Descriptor**	VdW + Hbond + desolv Energy			Moving Ligand-Moving Receptor		Internal Energy Receptor		Unbound System's Energy	
**Correlation**	0.17			0.26		-(0.098)		-(0.008)	

**V-life descriptors**									

**Descriptor**	chi6chain	chi5chain		SsBrE-index		T_2_F_1		T_N_F_7	
**Correlation**	0.42	-(0.54)		-(0.22)		0.36		0.39	

**Dragon descriptors**									

**Descriptor**	GATS4p	BELe1	H8v	R1p+	RTp+	nAr-CONR2	C-041	H-049	F-084
**Correlation**	0.27	0.38	0.31	-(0.55)	-(0.50)	0.35	-(0.13)	0.16	0.36

### Hybrid QSAR models

In this study, the best descriptors selected from different software like V-life, WEB-CDK, Dragon were combined and hybrid models were developed from these that encapsulated more information as compared to descriptors calculated from individual software. We developed three different types of hybrid models. Hybrid model 1 (Model 1) was developed using V-life and Web-Cdk descriptors and achieved r^2 ^= 0.60, which is better than individual models based on V-life or Web-Cdk descriptors (Table 2). Hybrid model 2 (Model 2) was build using descriptors obtained from V-life, Web-Cdk and docking energy and obtained r^2 ^= 0.63, which is significantly higher than r^2 ^of QSAR models individual descriptors. Third Hybrid model 3 (Model 3) was developed using V-life, Web-Cdk and Dragon based descriptors [See Additional file -3 for descriptor explanation] and achieved r^2 ^= 0.70, which is significantly better than any individual model [Table-3].

**Table 3:** The performance of QSAR models developed using descriptors calculated from two or more than two software packages.

Type of Model	Number of Descriptors	Software Packages	R	**R**^**2**^	MAE
Hybrid 1	7	V-life + Web-Cdk	0.77	0.6	0.33
Hybrid 2	11	V-life + Web-Cdk-Docking	0.79	0.63	0.32
Hybrid 3	15	V-life + Web-Cdk-Dragon	0.83	0.7	0.28

### Potential GlmU Inhibitors

#### Screening of Substrate similar Compounds

In this study, we predict chemical compounds that have the potential to inhibit GlmU target. We screened chemical libraries using QSAR models developed in this study. Firstly, a set of 15930 molecules were retrieved from PubChem having similarity more than 60% with GlmU substrate. We removed molecules that do not satisfy Lipinski rule of five. Finally we obtained 5008 molecules having 3D structural coordinates. These molecules were docked in binding pocket of GlmU using AutoDock (described in Receptor and Ligand preparation section) and docking energy was computed for each the molecule. Table 4, shows top 20 compounds having minimum docking energies, as shown energy varies from -9.84 to -8.73 along with inhibitory activity of these molecules.

**Table 4:** List of potential GlmU inhibitors selected based on minimum docking free energy

Substrate similar compounds				Anti-infective Compounds			
**S.No**.	**Compound ID**	**Free Energy of Binding**	**Predicted *IC50 *value**	**S.No**.	**Compound ID**	**Free Energy of Binding**	**Predicted *IC50 *value**

1	21681703	-9.84	82.94	1	4451056	-9.15	101.40
2	21597577	-9.37	109.93	2	4095801	-9.08	121.50
3	23421195	-9.27	82.94	3	702695	-8.87	85.30
4	24794354	-9.22	80.26	4	9612992	-8.74	126.62
5	21678408	-9.17	80.26	5	2236	-8.59	121.50
6	24794360	-9.17	80.26	6	3092	-8.36	111.76
7	24794349	-9.14	80.26	7	10751694	-8.12	111.77
8	21602943	-9.11	109.93	8	34318	-8.05	76.33
9	7098640	-9.03	80.26	9	5284340	-7.89	76.33
10	24794358	-9.02	80.26	10	93364	-7.65	66.27
11	21145106	-9.01	110.46	11	31715	-7.43	70.88
12	7098639	-8.97	109.93	12	39981	-7.35	101.40
13	24794356	-8.97	80.26	13	10611	-7.32	118.08
14	23421194	-8.94	82.94	14	2774	-7.3	118.08
15	20843309	-8.93	110.46	15	20824	-7.27	88.64
16	26470622	-8.92	109.93	16	7059498	-7.25	88.63
17	25202420	-8.9	109.93	17	3415	-7.22	101.40
18	23421196	-8.83	109.93	18	3070413	-7.21	127.04
19	21681821	-8.76	82.94	19	12874082	-7.21	70.89
20	4624316	-8.73	80.26	20	7018315	-7.19	61.59

These compounds were selected from group of compounds similar to substrate and anti-infective compound.

#### Screening of Anti-infective Compounds

We found a list of 3847 anti-infective compounds, out of which 1750 anti-infective compounds satisfy the Lipinski's rule. These compounds were retrieved from PubChem and used for screening against GlmU protein. These compounds were docked in the binding pocket of GlmU and docking energy was computed for each of the molecule. Based on minimum docking energy, we predicted 758 molecules as novel inhibitors of GlmU protein; top 20 compounds having minimum docking free energy is shown in Table 4. We also calculated inhibitory constant of these molecules using V-life descriptors based model.

The virtual screening of chemical compounds library predicts some potential inhibitors. Sometimes false positive prediction by docking or QSAR misleads thereby wasting time and money. Thus, it becomes difficult to identify a compound that is potentially active in experimental study. For example, in our case anti-infective compound PubChem ID 4451056 showed lower free energy as compared to compound PubChem ID 4095801 that is also in agreement with prediction by QSAR model. In such cases a hybrid approach could be beneficial. On this basis, we observed that there was a little difference in free energy of binding between compound 441056 and 4095801 and thus anti-infective compound 441056 could be used for experimental study having higher probability to act as potential inhibitor against GlmU enzyme.

#### Web Service to Community

One of the major objectives of our group is to bring down the cost of drug discovery. Unfortunately, most of the software for calculating molecular descriptors are commercial and come with number of restrictions. This webserver is a step to promote open source software in computer aided drug discovery. As shown in Table 3, we achieved best performance using model Hybrid 3. Unfortunately, Dragon is a commercial software come with restriction to use for public. Thus in this study, we developed a web server using second best model Hybrid 2, which used V-life, Web-Cdk descriptors and docking energies based descriptors. Though V-life is commercial software but we have license to use it for developing web services. Web-Cdk is based on CDK library which is open source. Server has been developed under Linux environment using CGI-Perl and Python scripts. In this web server, there are three options for molecule submission, 1) Draw structure using JME editor [31], 2) By pasting molecule in mol/mol2 file format, 3) By file upload using browse option. The result of prediction is seen interactively in the form of bound ligand in GlmU protein and its predicted *IC*_*50 *_value. We have also shown the descriptors used in this study along with Lipinski rule of five.

## Discussion

The trimeric GlmU protein is considered as a potential target for inhibitor design as it is essential for survival of bacteria. The identification of highly conserved amino acid residues from multiple sequence alignment reveals that single inhibitor may be able to kill wide range of bacterial species. The superimposition of *E.coli *structure 2OI6 and modeled *M. tuberculosis *structure shows rmsd of 1.02 A^0 ^using Matchmaker utility of chimera. Docking and QSAR are two well-known approaches in drug designing but each has its own limitation. While identification of lead molecules using QSAR techniques has been widely accepted in the absence of crystal structure of target molecule, docking based method is considered to be more accurate if the target structure is available. Thus we have used both techniques for predicting potential inhibitors.

Recently, a collaborative drug discovery program (CDD) [19] yielded a collection of potential anti tubercular compounds and predictive models for the same, but our study is focused on identification of potential inhibitors of GlmU using hybrid approach. In this study, a wide range of machine learning techniques has been used to develop QSAR models. It was found that MLR based model performs nearly equal/better as compared to other machine learning techniques. In order to avoid over optimization, it is important to follow (n < 4d) rule where number of descriptors should be less than one fourth of total compounds. All software calculates large number of descriptors, thus there is a need to reduce number of descriptors by removing irrelevant, duplicate and highly correlated descriptors so that we can narrow down to best-performing as well best-representative descriptor set. As shown in Table 2, V-life descriptor chi5chain, Web-Cdk descriptor VCH-4 and Dragon descriptor R1p+, Rtp+ high correlation >0.50 with *pIC*_50 _value, which demonstrate the importance of these descriptors. While among docking based descriptors, Moving Ligand-Moving Receptor shows maximum correlation 0.26 with *pIC*_50. _The better performance of dragon based selected descriptors may be due to the presence of two descriptors namely R1P+, RTP+ that shows high correlation with inhibitory activity as compared to other that have only one descriptor that shows high correlation. In this study, we integrated both QSAR and docking techniques for predicting inhibition potential of compounds. Using only docking energies as descriptors may give poor correlation because it's not always true that the pose with lowest binding energy is the one with the lowest RMSD and also practically impossible to analyze each docking pose. Besides, there are other kinds of interactions that play important role in predicting binding energies. Thus a hybrid approach may be beneficial to develop better predictive model. As shown in Table 3, hybrid method which combined two or more than two types descriptors. Based on this study, we have screened potential inhibitors against GlmU and predicted 40 compounds as potential inhibitor. By developing BioAssay using recombinant protein, validation of these inhibitors by others will confirm our algorithms and methodology. We hope our web service will serve the community involved in drug discovery as well as it will encourage other scientist working in the field of informatics to develop free software/web-servers.

## Conclusion

This study describes the development of a freely available webserver for screening chemical compounds library against GlmU protein. The docking approach also provides valuable information about protein-ligand interaction and help in further ligand based drug designing. This server will be useful to narrow down the time and cost required to screen a chemical library.

## List of Abbreviations

QSAR: Quantitative Structural Activity Relationship; GlmU: N-Acetyl-glucosamine-uridyltransferase; CDD: Collaborative Drug Discovery; MLR: Multiple Linear Regression; LOOCV: Leave-One-Out Cross-Validation; MAE: Mean Absolute Error; R: Correlation Coefficient; R^2^: Coefficient of determinant; SVM: Support Vector Machine; UDP-GluNAc: Uridine diphosphate N-Acetyl glucosamine-1-phosphate.

## Authors' contributions

DS developed QSAR models and perform site specific docking. MA and DD modeled GlmU protein of *M. Tuberculosis *and perform blind docking of inhibitors against modeled structure of GlmU. DS developed all QSAR models and web server. MA and DD edited the manuscript drafted by DS. GPSR conceived and coordinated the project as well as refined the manuscript. This manuscript has been seen and approved by all authors.

## Supplementary Material

Additional file 1 **Clustering of 125 inhibitors at threshold 0.7 using PubChem Clustering Tool**. Click here for file

Additional file 2 **Results of hybrid model on independent data sets**. Click here for file

Additional file 3 **Descriptors calculated from different software's with their explanation**. Click here for file
