# Supplementary figures and images for: A web server for predicting inhibitors against bacterial target GlmU protein
Source: BMC Pharmacol. 2011 Jul 6;11:5. doi: 10.1186/1471-2210-11-5 (PMC3146400; doi:10.1186/1471-2210-11-5)

**
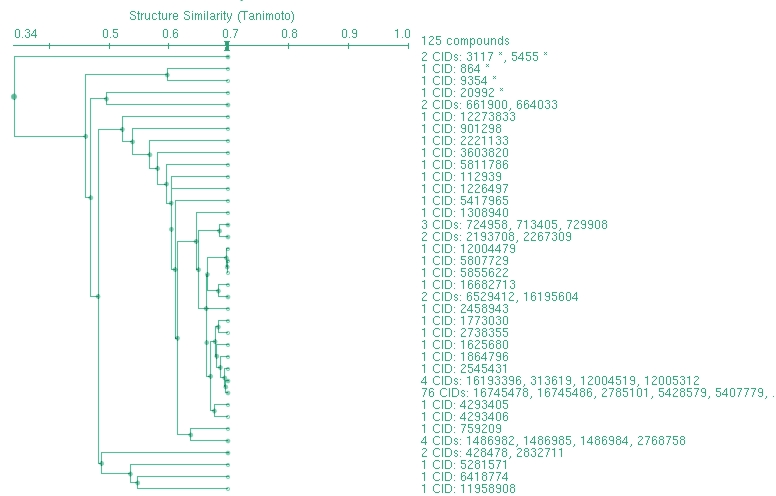
**

**Figure1 Shows the clustering of 125 inhibitors at threshold 0.7 using PubChem Clustering Tool**

Supplement: Additional file 1 — Clustering of 125 inhibitors at threshold 0.7 using PubChem Clustering Tool. [file 1471-2210-11-5-S1.DOC]
