# Supplementary material for: A web server for predicting inhibitors against bacterial target GlmU protein
Source: BMC Pharmacol. 2011 Jul 6;11:5. doi: 10.1186/1471-2210-11-5 (PMC3146400; doi:10.1186/1471-2210-11-5)
Supplement: Additional file 2 — Results of hybrid model on independent data sets. [file 1471-2210-11-5-S2.DOC]

**Table s1: shows the result in term of r and r^2^ of independent set and training set generated by random sampling method of hybrid method (V-life-WebCdk-Docking) based descriptors.**

| **Train Set** | | | **Independent Test Set** | |
| --- | --- | --- | --- | --- |
|  | **R** | **R^2^** | **R** | **R^2^** |
| 1 | 0.804 | 0.646 | 0.715 | 0.477 |
| 2 | 0.764 | 0.583 | 0.854 | 0.721 |
| 3 | 0.821 | 0.673 | 0.558 | 0.280 |
| 4 | 0.802 | 0.642 | 0.758 | 0.544 |
| 5 | 0.811 | 0.656 | 0.399 | 0.002 |
| 6 | 0.821 | 0.674 | 0.379 | 0.12 |
| 7 | 0.801 | 0.641 | 0.722 | 0.517 |
| 8 | 0.811 | 0.657 | 0.626 | 0.375 |
| 9 | 0.778 | 0.604 | 0.815 | 0.648 |
| 10 | 0.760 | 0.577 | 0.853 | 0.720 |
| 11 | 0.808 | 0.652 | 0.628 | 0.347 |
| 12 | 0.760 | 0.577 | 0.852 | 0.724 |
| 13 | 0.820 | 0.672 | 0.406 | 0.143 |
| 14 | 0.790 | 0.622 | 0.790 | 0.594 |
| 15 | 0.820 | 0.671 | 0.378 | -0.001 |
| 16 | 0.815 | 0.663 | 0.434 | 0.126 |
| 17 | 0.817 | 0.667 | 0.468 | 0.183 |
| 18 | 0.779 | 0.606 | 0.824 | 0.674 |
| 19 | 0.815 | 0.662 | 0.563 | 0.260 |
| 20 | 0.823 | 0.673 | 0.573 | 0.183 |
| 21 | 0.823 | 0.676 | 0.699 | 0.470 |
| 22 | 0.772 | 0.592 | 0.844 | 0.664 |
| 23 | 0.779 | 0.607 | 0.803 | 0.639 |
| 24 | 0.816 | 0.663 | 0.668 | 0.400 |
| 25 | 0.827 | 0.683 | 0.393 | 0.120 |
| **Average**  **0.801**   **0.641 0.64 0.397** | | | | |

**Table s2: shows the MLR result in term of r and r^2^ of independent set and training set generated by random sampling method of hybrid method (V-life-WebCdk-Drgon) based descriptors.**

| **Train Set** | | | **Independent Test Set** | |
| --- | --- | --- | --- | --- |
|  | **R** | **R^2^** | **R** | **R^2^** |
| 1 | 0.849 | 0.720 | 0.829 | 0.612 |
| 2 | 0.839 | 0.703 | 0.817 | 0.652 |
| 3 | 0.850 | 0.722 | 0.662 | 0.437 |
| 4 | 0.818 | 0.668 | 0.908 | 0.816 |
| 5 | 0.854 | 0.729 | 0.710 | 0.504 |
| 6 | 0.857 | 0.733 | 0.741 | 0.514 |
| 7 | 0.852 | 0.725 | 0.808 | 0.640 |
| 8 | 0.858 | 0.735 | 0.757 | 0.540 |
| 9 | 0.874 | 0.762 | 0.687 | 0.430 |
| 10 | 0.802 | 0.639 | 0.910 | 0.808 |
| 11 | 0.857 | 0.734 | 0.566 | 0.289 |
| 12 | 0.849 | 0.719 | 0.787 | 0.574 |
| 13 | 0.836 | 0.695 | 0.877 | 0.715 |
| 14 | 0.863 | 0.743 | 0.526 | 0.244 |
| 15 | 0.857 | 0.734 | 0.564 | 0.266 |
| 16 | 0.820 | 0.671 | 0.861 | 0.733 |
| 17 | 0.833 | 0.692 | 0.852 | 0.714 |
| 18 | 0.831 | 0.690 | 0.846 | 0.711 |
| 19 | 0.856 | 0.728 | 0.816 | 0.578 |
| 20 | 0.822 | 0.675 | 0.859 | 0.729 |
| 21 | 0.852 | 0.726 | 0.674 | 0.439 |
| 22 | 0.825 | 0.680 | 0.860 | 0.734 |
| 23 | 0.816 | 0.664 | 0.880 | 0.768 |
| 24 | 0.811 | 0.656 | 0.910 | 0.803 |
| 25 | 0.870 | 0.756 | 0.385 | 0.085 |
| **Average**  **0.842 0.708 0.763 0.573** | | | | |
