# Supplementary material for: A web server for predicting inhibitors against bacterial target GlmU protein
Source: BMC Pharmacol. 2011 Jul 6;11:5. doi: 10.1186/1471-2210-11-5 (PMC3146400; doi:10.1186/1471-2210-11-5)
Supplement: Additional file 3 — Descriptors calculated from different software's with their explanation. [file 1471-2210-11-5-S3.DOC]

**Table-s1: shows** the descriptors calculated from different software’s with their explanation

| **Descriptor Name** | **Explanation** |
| --- | --- |
| **Web-CDK descriptors** | |
| VCH-4 | Valence chain, order 4 (ChiChain) |
| Wlambda2.unity | Holistic descriptors |
| **V-life Descriptors** | |
| chi6chain | Retention index for five membered ring (Chi Chain) |
| chi5chain | Retention index for five membered ring (Chi Chain) |
| SsBrE-index | Electrotopological state indices for no. of bromine connected with one single bond (E-state contributions) |
| T_2_F_1 | Count no. of double bonded atoms seprated from any fluorine atom by 1 bond distance in a molecule (alignment independent descriptor) |
| T_N_F_7 | Count no. of Nitrogen atoms seprated from any fluorine atom by 7 bond in a molecule (alignment independent descriptor) |
| **Dragon Descriptors** | |
| GATS4p | Geary autocorrelation of lag 4 weighted by polarizability (2D autocorrelation) |
| BELe1 | Lowest eigenvalue n. 1 of burden matrix/weighted by atomic sanderson electronegativites (BCUT descriptor) |
| H8v | H autocorrelation of lag 8 / weighted by atomic van der Waals volumes (GETAWAY descriptor) |
| R1p+ | R maximal autocorrelation of lag 1 / weighted by atomic polarizabilities (GETAWAY descriptor) |
| RTp+ | R maximal index / weighted by atomic polarizabilities (GETAWAY descriptor) |
| nAr-CONR2 | No. of tertiary amide (aromatic) (Functional group counts) |
| C-041 | X-C(=X)-X (atom-centred fragments) |
| H-049 | H attached to C3(sp3) / C2(sp2) / C3(sp2) / C3(sp) (atom-centred fragments) |
| F-084 | F attached to C1(sp2) (atom-centred fragments) |
